# Novel role of BRCA1 interacting C‐terminal helicase 1 (*BRIP1)* in breast tumour cell invasion

**DOI:** 10.1111/jcmm.15761

**Published:** 2020-09-05

**Authors:** Balsam Rizeq, Saïd Sif, Gheyath K. Nasrallah, Allal Ouhtit

**Affiliations:** ^1^ Department of Biological and Environmental Sciences College of Arts & Sciences Qatar University Doha Qatar; ^2^ Biomedical Research Center Qatar University Doha Qatar; ^3^ Biomedical Science Department College of Health Sciences Qatar University Doha Qatar

**Keywords:** breast cancer, *BRIP1*, invasion, metastasis, proliferation, targeted therapy

## Abstract

Breast cancer (BC) is the most common malignancy and the leading cause of death in women worldwide. Only 5%‐10% of mutations in BRCA genes are associated with familial breast tumours in Eastern countries, suggesting the contribution of other genes. Using a microarray gene expression profiling study of BC, we have recently identified *BRIP1* (fivefold up‐regulation) as a potential gene associated with BC progression in the Omani population. Although *BRIP1* regulates DNA repair and cell proliferation, the precise role of *BRIP1* in BC cell invasion/metastasis has not been explored yet; this prompted us to test the hypothesis that *BRIP1* promotes BC cell proliferation and invasion. Using a combination of cellular and molecular approaches, our results revealed differential overexpression of *BRIP1* in different BC cell lines. Functional assays validated further the physiological relevance of *BRIP1* in tumour malignancy, and siRNA‐mediated *BRIP1* knockdown significantly reduced BC cell motility by targeting key motility‐associated genes. Moreover, down‐regulation of *BRIP1* expression significantly attenuated cell proliferation via cell cycle arrest. Our study is the first to show the novel function of *BRIP1* in promoting BC cell invasion by regulating expression of various downstream target genes. Furthermore, these findings provide us with a unique opportunity to identify *BRIP1*‐induced pro‐invasive genes that could serve as biomarkers and/or targets to guide the design of appropriate BC targeted therapies.

## 1 INTRODUCTION

Breast cancer (BC), a worldwide health problem, is the most common cancer in women, including the State of Qatar.^1^ In 2012, the number of new cancer cases was estimated to approximately 14.1 million, with 1.7 million BC cases diagnosed.^2^ An estimated number of 19.3 million new cases will be diagnosed each year by 2025.^3^ In the Gulf Cooperation Council (GCC) region, BC is also the most common cancer, and in Qatar, the rate of BC was 41% of all female cancer cases in 2013, with 56 per 100 000 as the risk of developing BC in the population (Qatar Cancer Registry, 2013). Curiously, the majority of BC cases present to the oncology clinic at the late stages of the disease with a particular tendency to affect younger ages.^4^, ^5^ The aetiology of BC in the region, including Qatar encompasses numerous risk factors such as late menopause, prolonged hormone replacement therapy, older age at first live childbirth, family history of BC at a young age and the mutations of the breast cancer associated gene 1 and 2 (*BRCA1/2)* genes.^4^, ^5^ New prognostic biomarkers need to be developed better‐targeted therapies against invasive stages of BC to enhance the chance for long‐term survival and patient's quality of life. To achieve this goal, it is imperative to understand the exact signalling pathways associated with the multistage process of metastasis.^6^ Invasion, the hallmark of malignancy, is the recurring and defining event in the metastatic process, and elucidation of its mechanisms is critical for developing effective anti‐metastatic therapies.

In GCC countries, where the rate of consanguinity is quite high (~50%), a significant number of BC patients are younger and show advanced tumours. In addition, a previous study conducted among Omani females with BC, showed no significant pathogenic BRCA1 gene missense mutations, suggesting the involvement of other genes in BC development. Based on the observations described above, we have previously used microarray analysis to compare RNA samples isolated from 40 malignant breast tumour tissues and 40 normal/benign breast tissues, and identified *BRCA1*‐interacting C‐terminal helicase 1 (*BRIP1*), showing fivefold induction, as a potential gene that might promote BC progression..^7^ *BRIP1*, also known as Fanconi Anaemia Group J Protein (*FANCJ)* or BRCA1‐associated C‐terminal helicase (*BACH1)*, was first identified using tandem mass spectrometry by its physical interaction with *BRCA1* and also belongs to the Fanconi anaemia (FA) genes family.^8^ *BRIP1* is located on chromosome 17q22, spanning a region greater than 180 kb, starting from 61 679 185 to 61 863 558 base pair with 20 exons and 19 introns.^8^ Interestingly, *BRCA1* is also located on chromosome 17q21 region, hence in close proximity with *BRIP1*.

*BRIP1* plays major role in DNA repair, type J Fanconi anemia, development of various cancers, including BC.^8^ BRIP1, a DNA‐dependent ATPase and a 5'‐3' helicase that belongs to the DNA‐dependent RecQ DEAH helicase family, interacts with BRCA1 and is involved in double‐stranded DNA breaks (DSB) repair during the G2‐M phase of the cell cycle as well as in tumour suppression.^9^, ^10^ *BRIP1* is expressed in both normal and malignant cells, and controls genome integrity *via* regulation of replication and homologous recombination,^11^ DNA damage responses and checkpoints, which are crucial for genomic stability.^9^ While *BRCA1* and *BRIP1* work as tumour suppressor genes,^10^ when *BRIP1* fails to bind *BRCA1* in certain conditions, cells become sensitive to different genotoxic stresses with aberrant homologous DNA repair function.^12^ Identification of *BRIP1* germline mutations in BC patient with wild‐type *BRCA1* and *BRCA2* suggests a major link between moderate penetrance of BC and *BRIP1* mutation.^13^, ^14^, ^15^, ^16^

Based on these observations, we hypothesized that beyond its function as a DNA repair gene, *BRIP1* plays a novel role in tumour cell invasion/metastasis. In addition, to providing a better understanding of the mechanisms that underpin *BRIP1*‐mediated tumour cell invasion, this study has validated *BRIP1* as a target gene that can be used to design efficient therapeutic strategies against BC.

## 2 MATERIALS AND METHODS

### 2.1 Cell culture

Human BC cell lines HCC‐2218, T47D, BT474, MCF‐7, CAMA‐1 and the immortalized human breast cell line MCF 10A were obtained from the American Type Culture Collection (ATCC). Immortalized HuMEC cells, MDA‐MB‐231 and MDA‐MB‐468 were provided by (Qatar University, Qatar), while HCC‐1500 cell line was obtained from (Weill Cornell Medical College, Qatar). HCC‐2218, T47D, BT474, MCF‐7 and CAMA‐1 cell lines were grown in DMEM medium (Gibco), while MDA‐MB‐231, MDA‐MB‐468 and HCC‐1500 cells were cultured in RPMI 1640 medium. Culture media were supplemented with 10% heat‐inactivated Foetal Bovine Serum (FBS) (Thermo Scientific) and 1% of an antibiotic suspension (Penicillin and streptomycin, Gibco). MCF 10A non‐pathogenic breast cell line was grown in HuMEC Basal serum‐free medium (Gibco, 12753018) supplemented with HuMEC Supplement Kit (Gibco, 12755013), while HuMEC cells were cultivated in keratinocyte serum‐free medium (SFM) supplemented with bovine pituitary extract (BPE) (Gibco, 17005042). All cells were maintained with an atmosphere of 5% CO_2_ in a humidified incubator adjusted to 37°C.

### 2.2 Western blotting

RIPA buffer supplemented with protease inhibitors (Pierce) was used to prepare whole cell lysates. Protein quantification was determined using Pierce™ BCA Protein Assay Kit (Thermo Scientific™) according to the manufacturer's detailed protocol. Approximately 20 µg of each denatured cell lysates was loaded on a 7.5% SDS‐PAGE. Electrophoresis was performed at 70V for 30 minutes and increased to 100V for 1‐2 hours in 1X running buffer then transferred into nitrocellulose membrane (Invitrogen). Subsequently, the membranes were blocked with 5% non‐fat dried milk in TBS/0.1% Tween 20 for 1 hour prior to probing with primary antibodies; Anti‐BACH1 rabbit affinity purified antibody (1:2500, B1310, Sigma) or monoclonal anti‐β‐Actin mouse antibody (1:2500, A2228, Sigma) overnight at 4°C. The membranes were probed with HRP‐conjugated anti‐rabbit secondary antibody (1:40 000 dilution, A0545, Sigma) to detect BRIP1 protein and anti‐mouse secondary antibody (1:2500 dilution, PAB0096, Abnova) to detect β‐Actin protein. The signal was revealed with SuperSignal™ West Pico PLUS Chemiluminescent Substrate (Thermo Scientific) and was developed using Chemiluminescent GeneGnome (Syngene). Densitometric quantitative analysis was performed using Image software (NIH Image Soft.).

### 2.3 Real‐time qRT‐PCR

According to the manufacturer's protocol, 1 μg of total RNA (extracted using GeneJET RNA Purification Kit (Thermo Fisher Scientific) was reversed transcribed into cDNA using the High Capacity cDNA Reverse Transcription Kit (Applied Biosystems) RT‐qPCR assay was performed to determine the expression of BRIP1 using TaqMan^®^ Fast Advanced Master Mix (Applied Biosystems). For each reaction, 1 uL of diluted cDNA (1:4) was added to 10 μL of TaqMan Master Mix, 1 μL of BRIP1 Gene Expression TaqMan^®^ Assays FAM‐MGB Probe (4351372, Applied Biosystems), and 1 μl of GAPDH Gene Expression TaqMan® Assays VIC‐MGB Probe (4448489, Applied Biosystems) as an endogenous control, and 7 μL of nuclease‐free water. The reaction was performed using the QuantStudio™ 6 Flex Real‐Time PCR System (Applied Biosystems, Inc) as follows: 10 minutes at 95°C (stage 1); 20 seconds at 95°C and 20 seconds at 60°C for 40 cycles (stage 2). The results were analysed using the QuantStudio™ 6 software.

### 2.4 siRNA transfection

Breast cancer cells were transfected with smart pool BRIP1 siRNA (50 nmol/L) (M‐010587‐00‐0010, Dharmacon Products) and Non‐Targeting siRNA Pool (si‐Ctrl) (D‐001206‐14‐05, Dharmacon Products) in Opti‐MEM medium (Invitrogen), using RNAi/Max Lipofectamine^®^ (Thermo Fisher Scientific) according to the manufacturer's protocol. Using four siRNA targeting different BRIP1 sequences lowers the likelihood of identifying off‐ or non‐specific targets in subsequent analyses (since the same off‐targets are not normally observed with two or more different BRIP1‐siRNAs). Cells were incubated for 24, 48 and 72 hours before gene expression analysis. In order to obtain effective gene silencing, the protocol has been standardized after several attempts, and a second transfection performed 24 hours after the first one showed better results. Different siRNA concentrations ranging from 30 to 100 nmol/L were tested, and 50 nmol/L concentration at 72 hours was selected as the optimal concentration showing the highest *BRIP1* silencing. Following incubation of the cells with variable siRNA concentrations, the cells were further analysed for gene expression, cell proliferation and cell motility.

### 2.5 Functional assays

#### 2.5.1 Cell proliferation assay

To explore the effect of *BRIP1* knockdown on proliferation rate in BC cell lines, Alamar Blue assay was performed according to the manufacturer recommendations (Invitrogen). Briefly, 10 000 cells of BRIP1‐siRNA and si‐Ctrl‐transfected cells were seeded 48 hours after siRNA transfection/100 µL per well in 96‐well plates (flat bottom Corning^®^ Costar^®^ cell culture plates). Next day, 10% of Alamar Blue reagent was added to each well after washing the cells twice with PBS, and the cells were incubated at 37°C for 4 hours in a dark chamber. Absorbance was measured at wavelengths 570 and 600 nm using BioTek Epoch2 (Synergy Multi‐Mode Reader, Inc). The results are presented as percentage difference in reduction between siRNA‐BRIP1 and si‐Ctrl‐transfected cells.

#### 2.5.2 In vitro cell invasion and migration assays

Cell migration and invasion assays were performed as we have previously described.^17^, ^18^ Briefly, for migration assay, 3 × 10^5^ of MDA‐MB‐231 or 5 × 10^5^ of (MCF‐7 and CAMA‐1) BRIP1‐siRNA and si‐Ctrl‐transfected cells were added directly to 8 mm PEC Transwell (without matrigel) chambers (Corning) for migration assays. For invasion assay, (matrigel coated) chambers (Corning) were initially incubated at 37°C for 2 hours. After incubation, transfected cells of 1 × 10^5^ of MDA‐MB‐231 or 10 × 10^5^ of (MCF‐7 and CAMA‐1) were added to the upper matrigel coated Transwell chambers. A volume of 650 μL media, containing 10% FBS as a chemoattractant, was added to the lower chamber. Cultures were maintained for 24 hours for MDA‐MB‐231 and 48 hours for MCF‐ 7 and CAMA‐1. For both migration and invasion assays, membranes were washed, fixed in 4% paraformaldehyde for 10 minutes and stained with 5% crystal violet in ethanol. Non‐penetrated cells were removed from the upper chambers using cotton swabs, and pictures were taken from five different microscopic fields using Cell Imaging System (Olympus IX73 inverted microscope). Fields were quantified and analysed using ImageJ software (NIH Image Software).

#### 2.5.3 Wound healing assay

Briefly, MCF‐7, CAMA‐1 and MDA‐MB‐231 cells were transfected with BRIP1 siRNA or non‐targeted siRNA (si‐Ctrl). Then, the cells were counted and seeded in a 24‐well plate with serum‐free media. A straight scratch was made in each well using a sterile 10 μL white tip. Cells were washed gently with sterile PBS to remove debris and then cultured in their corresponding complete growth media. The plates were then incubated and photographs were taken at 0, 24 and 48 hours, depending on the cell line used in reference to a marker line for accurate imaging. Results were analysed using the ImageJ software (NIH Image Software).

#### 2.5.4 Cell cycle

Cell cycle analysis was carried out using fluorescence‐activated cell sorting (FACS) method. In Both siRNA‐BRIP1 and si‐Ctrl‐transfected, cells were trypsinized using TrypLE reagent (Gibco), fixed and incubated overnight at 4°C. Next day, the cells were centrifuged, washed and then incubated with 40 μg/mL propidium iodide (PI) and 100 μg/mL RNase (Sigma) in the dark at room temperature for 30 minutes. Stained cells were then analysed by BD Accuri™ C6 Flow Cytometer (BDbiosciences). Non‐stained and PI‐stained cells were used as controls. Cell cycle distribution analysis was determined using (FlowJo™ v10.6.1, LLC) software.

### 2.6 Metastasis‐associated genes profiling

Predesigned tumour Metastasis Fast 96‐well plates (4414098, Life Technologies) containing lyophilized TaqMan^®^ Gene Expression assay was used to determine the expression levels of pro‐metastatic genes in BRIP1‐siRNA transfected cells compared with si‐Ctrl. Briefly, total RNA was converted into cDNA as described above. The metastasis‐associated genes profiling reaction was performed using QuantStudio™ 6 Flex Real‐Time PCR System (Applied Biosystems, Inc), according to the manufacturer instructions. The results were first normalized to GAPDH and further analysed to obtain the Relative mRNA expression levels, using the formula 2^−ΔΔCt^. All the reactions were carried out in triplicates and repeated twice.

### 2.7 Statistical analysis

In proteomics analysis, Student's two‐tailed test was used to assess the statistical significance of the protein quantification. Statistical significance between groups was assessed using ANOVA tests and Student's *t* test. For other experiments, a non‐parametric Student's *t* test was applied using Microsoft Excel 2013 and GraphPad Prism 8. Data of three independent experiments were presented as means ± SD unless otherwise indicated in the legends. *P* values ≤.05 were considered statistically significant.

## 3 RESULTS

### 3.1 BRIP1 is highly expressed in different breast cancer cell lines

**FIGURE 1:**
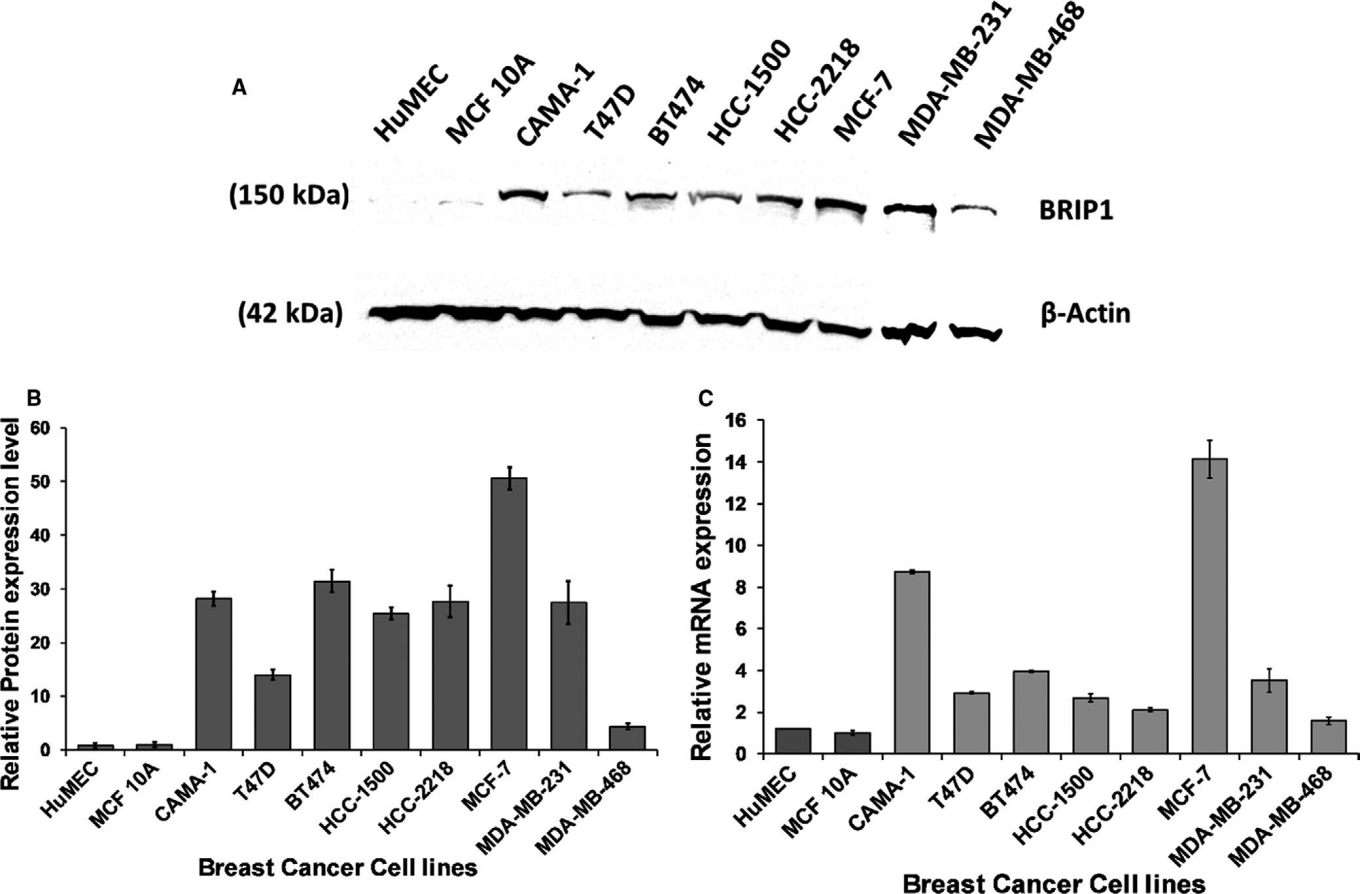
BRIP1 protein and mRNA expression levels in various BC cell lines. (A) Western Blot analysis showing BRIP1 protein expression levels in various BC cell lines. β–Actin was used as loading control. Shown are representative immunoblots from the same experiment, repeated three separate times under the same experimental conditions. (B) Relative expression levels of BRIP1 protein in various BC cells determined by densitometry. (C) TaqMan RT‐qPCR relative expression levels of BRIP1 mRNA normalized to GAPDH. The data are represented as mean ± SD (n = 3)

Both *BRIP1* protein and mRNA levels were differentially overexpressed in various BC cell lines compared with control HuMEC and MCF 10A cells (Figure 1). At the protein level, Western blotting analysis followed by densitometric quantification showed relative overexpression of BRIP1 compared with control (Figure 1B). Similarly, BRIP1 mRNA levels showed 1.8 to 14‐fold increase in the tested BC cell lines compared with normal/control cells (Figure 1C).

### 3.2 Down‐regulation of BRIP1 using siRNA interference in breast cancer cell lines

**FIGURE 2:**
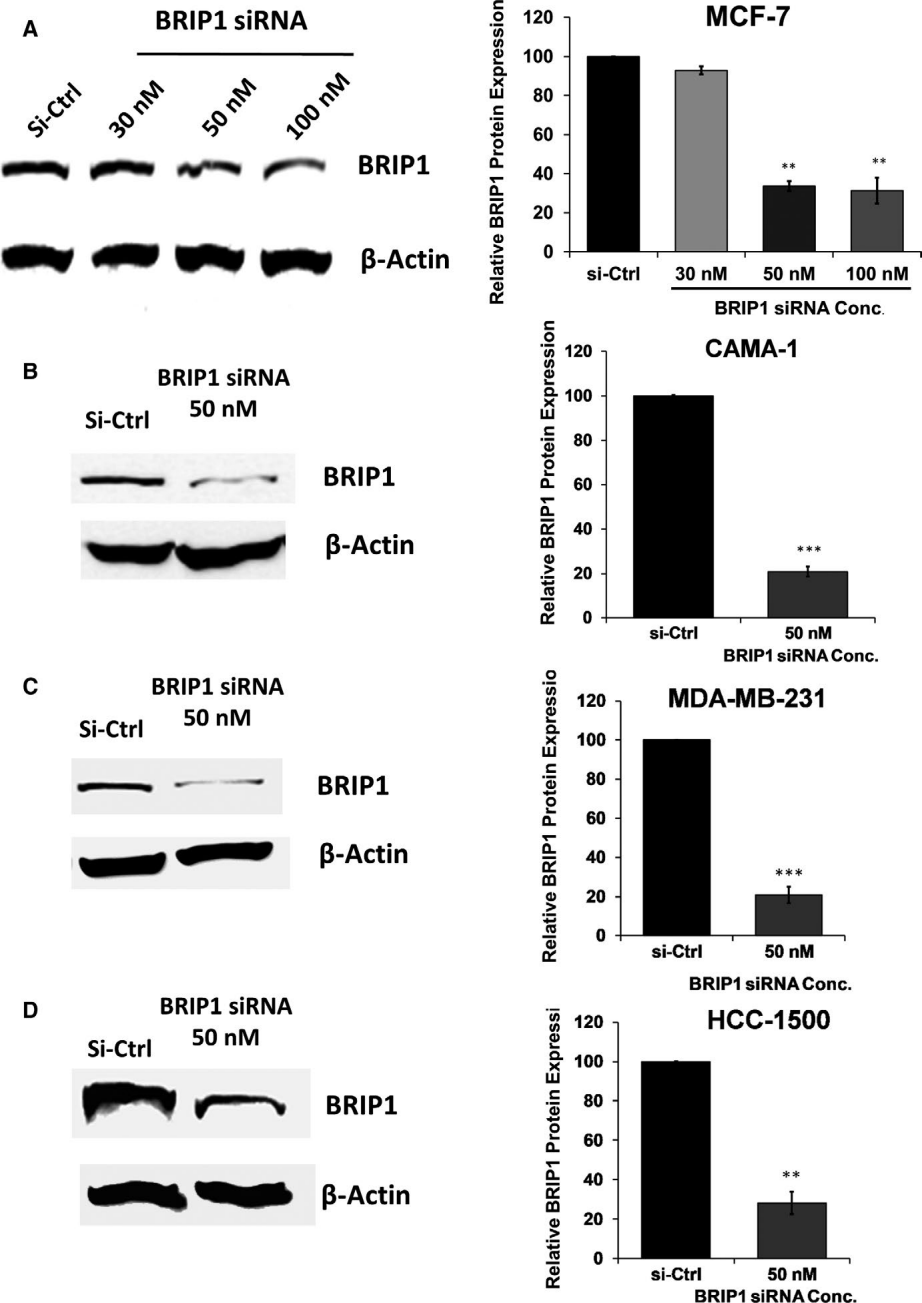
Protein expression levels of BRIP1 in BC cells transfected with a pool of four siRNA targeting different specific BRIP1 sequences. Representative Western blot analysis of BRIP1 and β‐Actin protein expression after siRNA transfection compared with si‐control in (A) MCF‐7 cells; (B) CAMA‐1 cells; (C) MDA‐MB‐231 cells; and (D) HCC‐1500 cells. Shown are representative immunoblot images from three separate experiments, which were performed under the same experimental conditions. Each band was measured by densitometry and normalized to the corresponding β‐Actin control. The results were normalized to the control (si‐Ctrl). The data are represented as the mean ± SD (n = 3); *P < .05, **P < .01, ***P < .001

Four different Dhramacon SMARTpool siRNAs specific to BRIP1 were mixed, providing both specificity and potency advantages in inhibiting BRIP1 expression in BC cells. The siRNA concentration that resulted in the most efficient silencing of BRIP1 with the least cell toxicity was selected for all experiments. Treatment of selected BC cells with increasing concentrations of siRNA mixture ranging from 30 to 100 nmol/L showed the highest decrease in BRIP1 protein levels by approximately 70% with 50 nmol/Lo at 72 hurs in MCF‐7 (Figure 2A). The dose of 50 nmol/L was selected for all other three cell types, showing 80% reduction of BRIP1 in CAMA‐1 and MDA‐MB‐231 (Figure 2B‐C), respectively, and 70% inhibition in HCC‐1500 (Figure 2D). *BRIP1* mRNA levels were significantly decreased in all transfected BC cells compared with si‐Ctrl‐transfected cells (see Figures S1 and S2 in Supplementary Data).

### 3.3 BRIP1 facilitates proliferation in breast cancer cell lines

**FIGURE 3:**
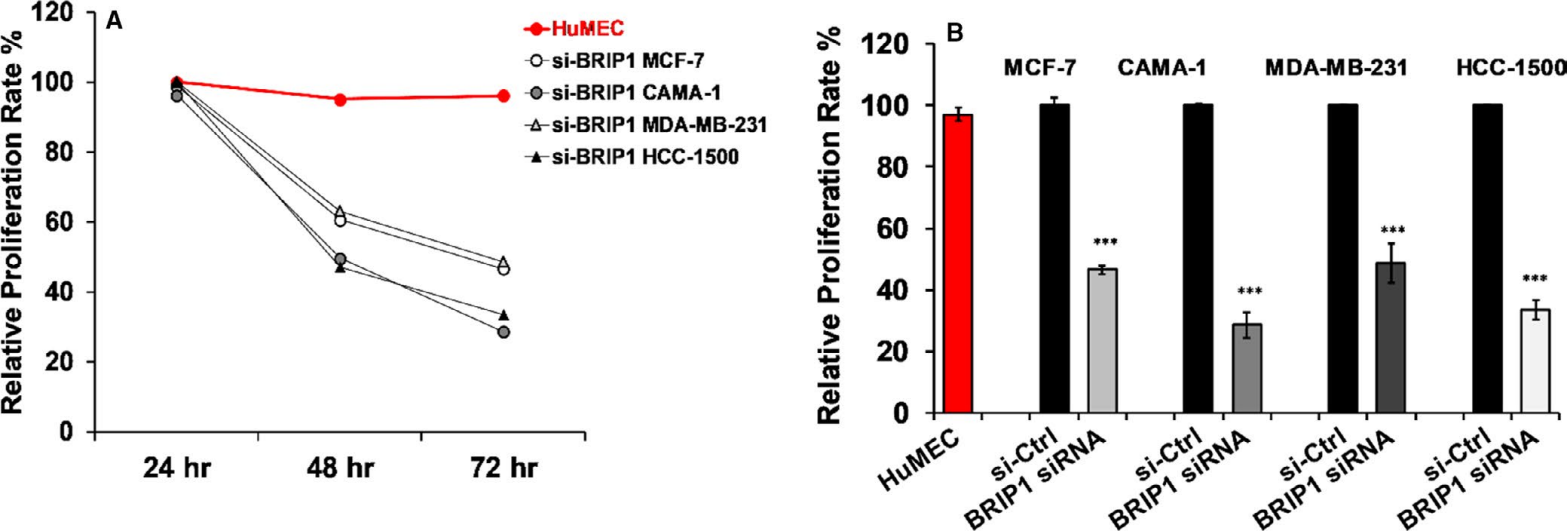
Effect of BRIP1 siRNA suppression on cell proliferation in various breast cancer cell lines. (A) Relative cell proliferation rate at 24, 48 and 72 h post‐transfection with the pool of BRIP1‐specific siRNAs (50 nmol/L) in MCF‐7, CAMA‐1, MDA‐MB‐231 and HCC‐1500 breast cancer cell lines. (B) All tested si‐BRIP1 BC cell lines showed maximal inhibition in proliferation 72 h post‐treatment. The data represent analysis of results from replicate experiments using the mean ± SD (n = 3). ***P < .001

**FIGURE 4:**
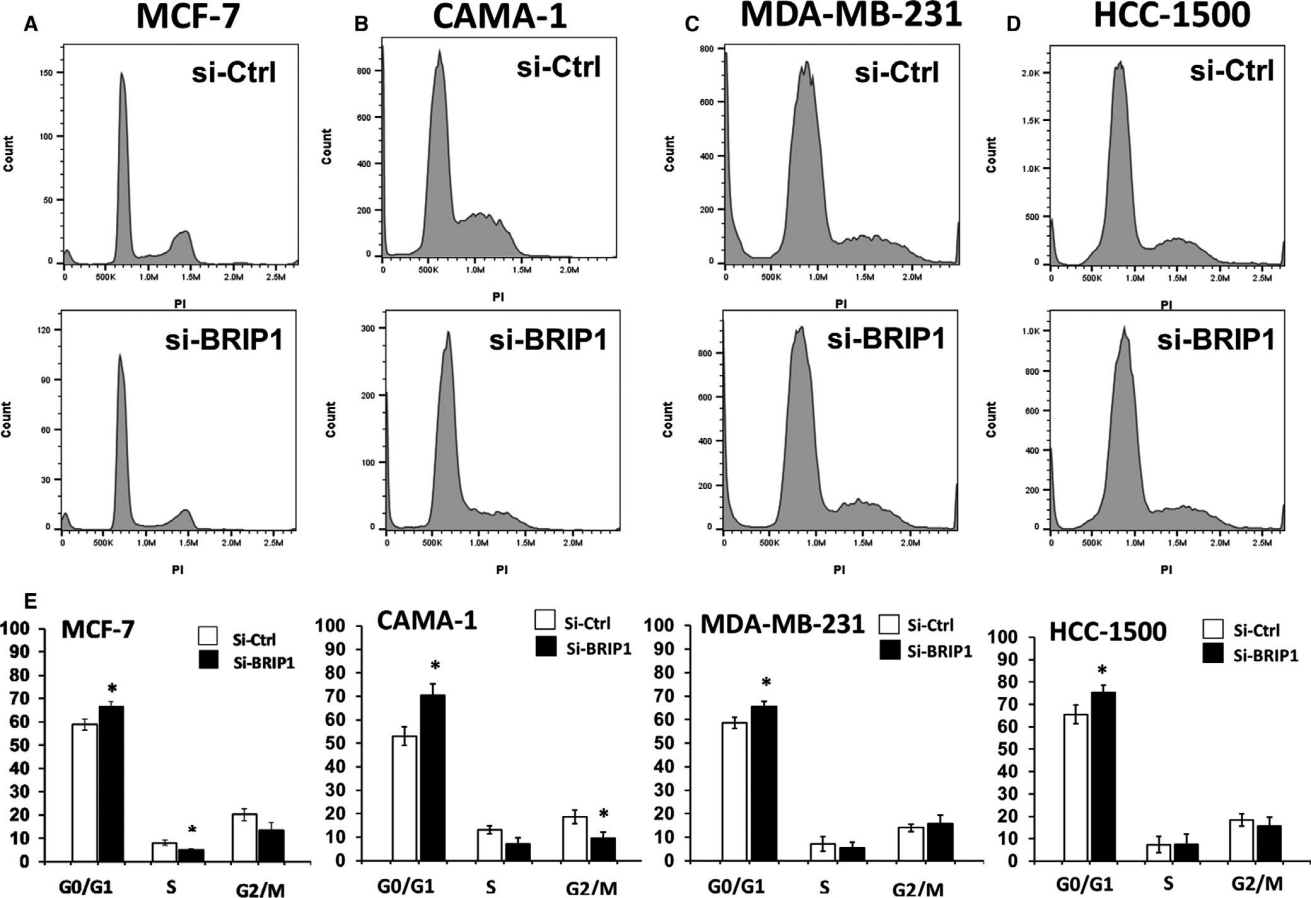
si‐RNA suppression of BRIP1 induced cell cycle arrest in various BC cells. Representative analysis of Cell cycle phases in (A) MCF‐7, (B) CAMA‐1, (C) MDA‐MB‐231 and (D) HCC‐1500 BC cells treated with BRIP1‐siRNA compared with si‐Control transfected cells at 72 h using flow cytometry. (E) Representative qualitative analysis of G0/G1, S, and G2/M cell cycle phases of all BRIP1‐siRNA transfected BC cell lines compared with si‐Ctrl. The quantitation was done by calculating the area under the curve using Flowjo software and is presented as the mean ± SD for three independent experiments, *P < .05

To examine the effect of *BRIP1* inhibition on BC cell proliferation, cells were treated with either BRIP1‐siRNA or si‐Ctrl, and cellular proliferation was assessed at 24, 48, and 72 hours post‐treatment using Alamar blue assay. As shown in Figure 3, cellular proliferation was significantly reduced in si‐BRIP1 transfected cells compared with si‐Ctrl groups in all studied BC cell lines. Despite a slight variation between the different BC cell lines, there was a decrease by 46.5% in MCF‐7, 28.6% in CAMA‐1, 48.6% in MDA‐MB‐231 and 33.5% in HCC‐1500 72 hours post‐treatment (Figure 3B). Fluorescence‐activated cell sorting (FACS) analysis revealed that *BRIP1* suppression induced G1/S arrest in all the studied si‐BRIP1‐transfected BC cells (Figure 4). All the data put together indicate that *BRIP1* promotes BC cell proliferation.

### 3.4 BRIP1 promotes migration and invasion ability of breast cancer cell lines

**FIGURE 5:**
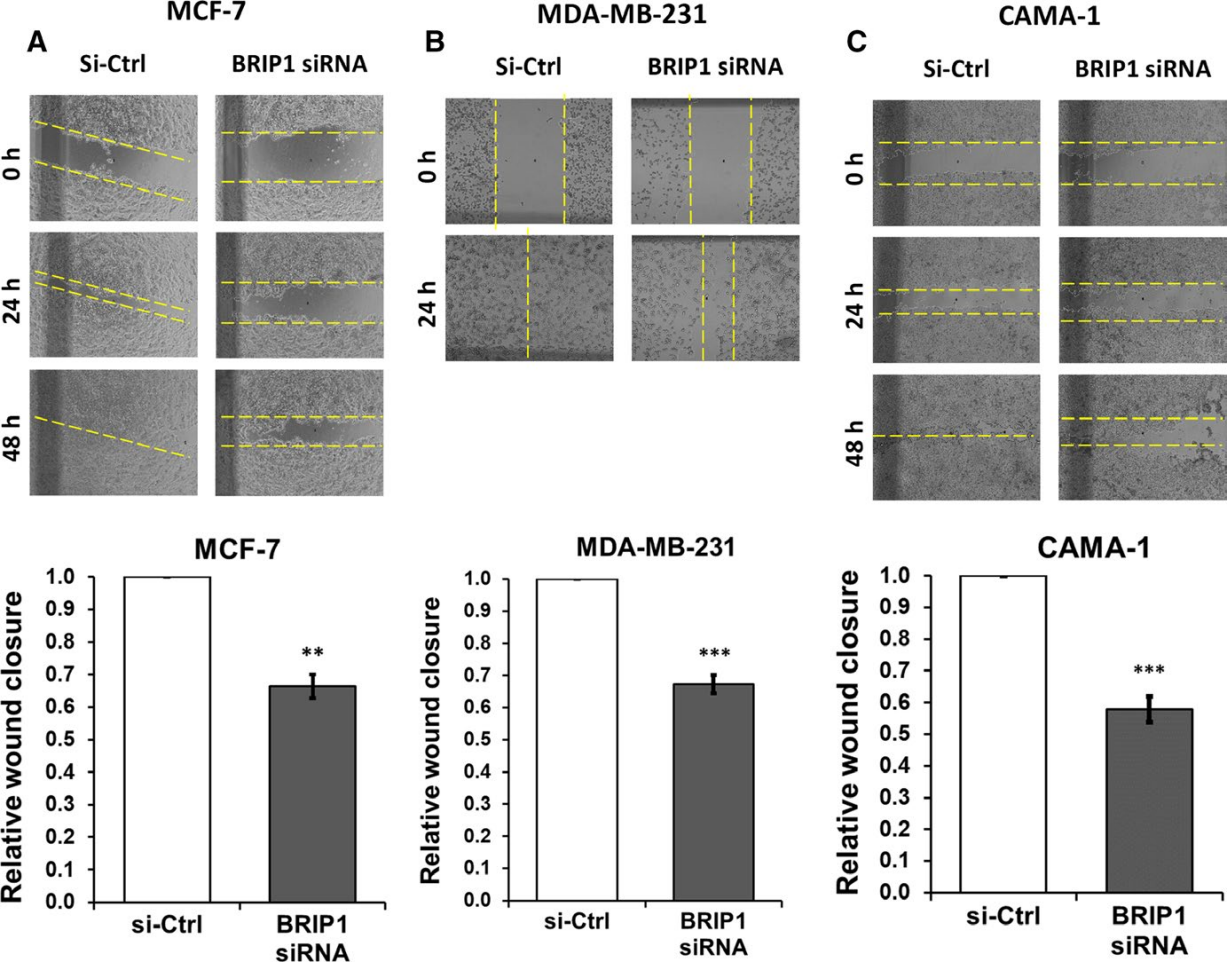
Wound‐healing assay showing the effect of BRIP1 siRNA inhibition on cell migration in selected breast cancer cell lines (A) MCF‐7 (B) MDA‐MB‐231, and (C) CAMA‐1. Graphical representation of the relative wound closure following BRIP1‐specific siRNA treatment compared with si‐Ctrl at 48 h in MCF‐7 and CAMA‐1, and at 24 h in MDA‐MB‐231. Data shown are representatives of three independent experiments under the same conditions. The error bars represent the SD. **P < .01, ***P < .001

**FIGURE 6:**
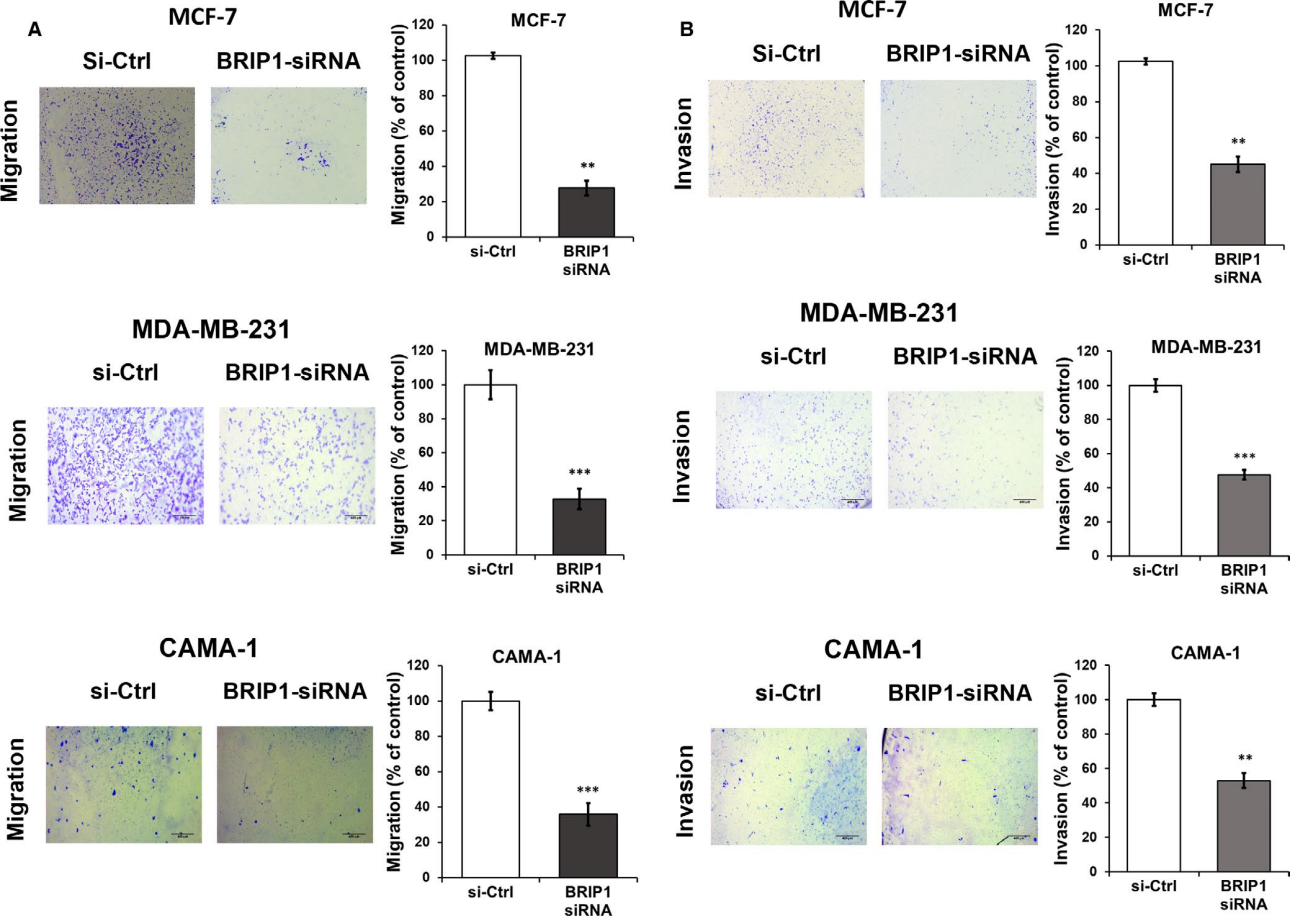
BRIP1 suppression decreases BC cells' migration and invasion. Boyden chamber assay was used to assess both cell migration and invasion in MCF‐7, MDA‐MB‐231 and CAMA‐1 BC cells following BRIP1‐siRNAs and si‐Ctrl (control group) transfection. (A Panel) showing crystal violet‐stained migrated cells at the lower part of the transwell chamber membrane following BRIP1‐specific siRNA transfected in MCF‐7, MDA‐MB‐231 and CAMA‐1 BC cells. (B Panel) showing crystal violet‐stained invaded cells at the lower part of the Transwell chamber membrane following BRIP1‐specific siRNA transfected in MCF‐7, MDA‐MB‐231 and CAMA‐1 BC cells. The relative percentage of migrated (A) and invaded (B) cells after knockdown with si‐BRIP1 compared with control showing that BRIP1 suppression decreased both migration and cell invasion compared with si‐Ctrl group. The data represent the relative number of transfected cells with a minimum of three fields randomly selected to count cells. (n = 3, **P < .01, ***P < .001)

Wound healing assay results revealed that *BRIP1* suppression significantly attenuated the ability of cells to close the gap. Compared with control cells, a relative wound closure of 60% in MCF‐7 and MDA‐MB‐231, and 50% in CAMA‐1 was observed (Figure 5). Furthermore, in non‐coated Transwell assay, compared with the control groups, the migration ability of si‐BRIP1‐inhibited cells was significantly reduced by 70% in MCF‐7 and MDA‐MB‐231, and 60% in CAMA‐1 (Figure 6A). Suppression of *BRIP1* showed significant inhibition of invasion by 50% in MCF‐7, MDA‐MB‐231 and CAMA‐1 compared with si‐Ctrl‐transfected cells (Figure 6B). Together, our results indicate that suppression of *BRIP1* inhibited both migration and invasion in all tested BC cell lines.

### 3.5 BRIP1 promotes cell invasion by modulating expression of an array of related genes

In order to identify potential molecular signalling pathway(s) underpinning the novel role of *BRIP1* in BC progression, TaqMan RT‐qPCR of 92 different metastasis‐associated genes was performed to determine differentially expressed genes in *BRIP1*‐suppressed MCF‐7 and MDA‐MB‐231 cells compared with controls. Analysis of our results showed a significant up‐regulation/down‐regulation of a group of genes known to regulate both cell growth and cell motility. Among several differentially expressed genes, the present study focused on genes associated with extracellular matrix, adhesion molecules, cell proliferation and motility (C‐X‐C motif chemokine 12 [*CXCL12], Retinoblastoma 1 [RB1], Ras Homolog Family Member C [RHOC], H/K Retrovirus‐Associated DNA Sequences [H/KRAS], Epithelial cell adhesion molecule [EPCAM], Avian myelocutomatosis viral oncogene homolog [MYC], SMAD family member 4 [SMAD4], Melanoma Cell Adhesion Molecule [MCAM [CD146]], Alpha‐1,6‐Mannosylglycoprotein 6‐Beta‐N Acetylglucosaminyltransferase [MGAT5]* and Matrix Metalloproteinases [*MMPs]*) was determined (Table 1).

**TABLE 1:** BRIP1 promotes cell growth and metastasis by modulating the expression of an array of related genes

Symbol	TaqMan Assay ID	Description/Main function	Fold change in BC cells^siRNA‐BRIP1^ vs BC cells^si‐Ctrl^)
*CXCL12*	Hs00171022_m1	C‐X‐C Motif Chemokine Ligand 12/Tumour growth and metastasis	−5.8
*RB1*	Hs00153108_m1	RB Transcriptional Corepressor 1/Cell growth and cell cycle	−4.8
*RHOC*	Hs00733980_m1	Ras Homolog Family Member C/Tumour cell proliferation and metastasis.	−3.9
*HRAS*	Hs00610483_m1	HRas Proto‐Oncogene, GTPase/Regulation of cell proliferation	−3.8
*MYC*	Hs00153408_m1	MYC Proto‐Oncogene, BHLH Transcription Factor/cell cycle progression, apoptosis and cellular transformation	−3.7
*SMAD4*	Hs00232068_m1	SMAD Family Member 4/Cell proliferation.	−3.5
*KRAS*		KRAS Proto‐Oncogene, GTPase/Regulation of cell proliferation	−3.0
*EPCAM*	Hs00158980_m1	Epithelial Cell Adhesion Molecule/Proliferation, differentiation and migration.	−2.9
*MCAM* (*CD146*)	Hs00174838_m1	Melanoma Cell Adhesion/cell adhesion Molecule/Cell adhesion	−2.9
*MGAT5*	Hs00159136_m1	Alpha‐1,6‐Mannosylglycoprotein 6‐Beta‐N‐Acetylglucosaminyltransferase/Proliferation, adhesion and metastasis	−2.5
*MMP‐1*	Hs00233958_m1	matrix metallopeptidase 1/Breakdown of extracellular matrix and metastasis	−2.0

Note

The list of selected genes analysed using the TaqMan™ Array Human Tumour Metastasis (Applied Biosystems™). Fold change was calculated by comparison of Comparative Ct values for siRNA‐BRIP1 transfected cells compared with si‐Ctrl cells for three biological replicates. Statistical analysis was performed using Student's *t* test. Genes with fold changes below 2 or above −2 (Cut‐off point) and/or when the *P*‐value >.05 were not considered in the table.

## 4 DISCUSSION

We have previously employed microarray gene expression profiling and compared RNA samples isolated from malignant breast tumour tissues to normal/benign breast tissues.^7^ Among a number of differentially expressed genes, *BRIP1*, showing a fivefold up‐regulation, was identified as a potential gene that might underpin breast tumour progression.^7^ Although BRIP1 interacts with BRCA1 to regulate cell cycle and DNA repair mechanisms, the role of *BRIP1* in mediating tumour growth and progression has not been examined yet. In the present study, we tested the hypothesis that *BRIP1* can promote both BC cell growth and metastasis. The results of the present study are summarized as follows: (a) structural validation experiments were consistent with our previous findings,^19^ showing fivefold overexpression of *BRIP1* in all types of BC tumour samples compared with normal/benign breast tissues; (b) functional validation approaches revealed a novel role of *BRIP1* in promoting breast tumour cell growth and progression. In fact, siRNA down‐regulation of *BRIP1* attenuated significantly cell proliferation by inducing cell cycle arrest. Moreover, RNAi‐inhibition of *BRIP1* significantly reduced migration and invasion of BC cell lines; (c) a unique set of differentially expressed *BRIP1*‐target genes associated with both cell cycle and metastasis seem to underpin *BRIP1*‐promoted BC cell proliferation and invasion.

### 4.1 BRIP1 overexpression in breast cancer

The mutational spectrum of *BRIP1* was recently investigated in various BC cell lines using the *Estimate algorithm of* genome‐wide copy number analysis and hybrid capture sequencing of 1651 genes ^20^ showed no BRIP1 mutations in our selected model for functional characterization. Up‐regulated *BRIP1* levels in malignant breast tumours contradict its role as a tumour suppressor. As an example, the tumour protein 53 (*TP53)* tumour suppressor gene is overexpressed in colorectal cancer, which is not predictive with its mutational status as an early event,^21^ suggesting that *TP53* has an oncogenic role independent of the tumour suppression activity. This dual behaviour was also reported for other genes, such as Wilms’ tumour 1 (*WT1*); mutated *WT1* led to the onset of kidney tumours, and its overexpression was detected in a subset of human cancers.^22^

Although gene amplification plays a major role in tumorigenesis, especially for solid tumours (reviewed in,^23^, ^24^ the molecular mechanisms of how these genes are amplified are not fully understood yet. Plausible mechanisms of gene amplification may include episome excision,^25^ re‐replication and unequal exchange.^26^

Bioinformatic analysis of approximately 2000 BC patients, revealed that the 17q23 region was associated with the presence of oncogenes,^27^ BC poor prognosis and tumour progression.^27^, ^28^ Gain of function resulting from amplification of this region has been reported in other cancers, including liver,^29^ pancreas,^30^ bladder,^31^ testis,^32^ ovary,^33^ lung ^34^ and brain tumours.^35^ Interestingly, the 17q23 amplicon where *BRIP1* gene lies, encompasses known oncogenes *RPS6KB1*, *TBX2* and *PPM1D*.^35^ In particular, TCGA showed a 3.2‐fold up‐regulation of *BRIP1* in breast tumours compared with normal breast tissues.^36^ Similarly, our microarray data revealed that *BRIP1* had an average of fivefold overexpression as compared to normal breast tissue samples.^7^

Consistent with these observations, our results showed clearly that both the protein and mRNA levels of BRIP1 were overexpressed in various BC cell lines compared with control normal/immortalized and normal/control breast cells, suggesting that *BRIP1* might act as an oncogenic driver in BC. Interestingly, previous study revealed the presence of *BRIP1* germline mutations in BC patients without any *BRCA* mutation, suggesting a link between *BRIP1* mutations and BC susceptibility.^19^ Collectively, although *BRIP1* is considered as a tumour suppressor gene, it is amplified in sporadic cancers,^36^ thus supporting our previous ^7^ and current findings, suggesting that *BRIP1* amplification in sporadic cancers could explain the oncogenic role of BRIP1.

### 4.2 BRIP1 promotes breast cancer cell proliferation

In order to understand the mechanisms that underpin *BRIP1*‐promoted BC cell proliferation, our results revealed that siRNA *BRIP1* suppression, arrested cell cycle at the G1/S phase, and TaqMan array identified several key cell cycle regulators that were down‐regulated upon BRIP1 inhibition, including *c‐Myc* (−3.7), *Ras GTPase* (−3.8) and *Rb* (−4.8) accompanied by cell cycle arrest at G1.^37^ In G1/S, Ras‐activation drives Myc accumulation and regulates proliferation‐related genes through E2F transcriptional activity.^37^, ^38^ Normally, *c‐Myc* is expressed during active cell division phase only, regulates the transition of cells from G1 to S phase and is associated with poor prognosis ^39^ (Reviewed in ^40^). RNAi‐inhibited *c‐Myc* reduced MCF‐7 BC cells by 30% as well as tumour development in nude mice.^41^ Although our results showed that inhibition of BRIP1 reduced RB expression levels, ongoing experiments aim to evaluate the expression levels of phosphorylated Rb, expected to decrease with increased BRIP1 expression to arrest cell cycle. However, during BC metastasis, Rb is expected to increase to promote cell growth and invasion. *Ras GTPase* pathway appears to be required in all decisions during both G1 and G2 phases.^42^ Ongoing pharmacological and functional approaches aim to further validate whether these genes underpin BRIP1‐promoted cell growth.

### 4.3 BRIP1 promotes breast tumour cell invasion

On the other hand, and to our knowledge, no study has linked *BRIP1* to cancer metastasis yet. Our siRNA experiments suggest that *BRIP1* up‐regulation may promote breast tumour metastasis. In fact, previous studies revealed that overexpression of RhoA GTPase was associated with carcinogenesis.^43^ In the present study, RNAi‐inhibited *BRIP1* down‐regulated *Ras GTPases* significantly by (−3.9) fold and subsequently suppressed tumour cell invasion.

Furthermore, si‐BRIP1 has markedly down‐regulated *MMP‐1* (−2.1 fold), a member of endopeptidase zinc‐dependent family that cleave the extracellular matrix.^44^ *MMP‐1* down‐regulation significantly attenuated cell proliferation, migration and invasion and reduced the expression of *Myc* in MCF‐7 and MDA‐MB‐231 cells.^45^ In addition, *BRIP1* might contribute to BC bone metastasis by switching the SMAD pathway; (SMAD4 was down‐regulated by (−3.5) fold) from the known tumour suppression role to pro‐metastatic one.^46^

*MGAT5* gene is another BRIP1‐transcriptional target gene that was down‐regulated upon siRNA inhibition of BRIP1 (−2.5 fold). In fact, *MGAT5* overexpression has been reported in various human cancers, including hepatocarcinoma, colon cancer and BC.^47^, ^48^, ^49^ *MGAT5* is known to promote both cell growth, cell motility,^50^, ^51^ leading to tumour progression. A recent study demonstrated that restoration of *MGAT5* expression in MCF‐7 and MDA‐MB‐231 overcame the inhibitory effect of miR‐124 on BC cell progression.^52^

Also, our results showed that *BRIP1* down‐regulation reduced the expression of *CXCL12* significantly (−5.8 fold). Structure‐function studies showed that CXCL12 chemokine is the only ligand for CXCR4, and thus activation of CXC4 depends on the CXCL12‐induced chemokine.^53^ Attenuation of either one of them, using neutralizing antibodies, reduced angiogenesis and metastasis.^54^

Finally, genes coding for cell adhesion molecules, known to mediate BC progression, were also identified in our Taq Man experiments, including *EPCAM* (−2.9 fold change) and *MCAM* known as CD146 (‐ 3 fold change). While, *EPCAM*, which is overexpressed in most human carcinomas, abrogates E‐cadherin‐mediated cell‐cell interaction,^55^ *MCAM* overexpression is associated with increased expression of epithelial‐mesenchymal transition (EMT) biomarkers and cell motility.^56^

In conclusion, BRCAness status and family history support the idea of the association of other moderate/low penetrance genes in the onset of BC, such as BRIP1, which binds to BRCA1 to maintain genome integrity.^3^ The present study revealed a novel role of BRIP1 in promoting BC cell growth and invasion. In addition, a number of BRIP1‐transcriptional target genes associated with cell growth and cell invasion were identified. While these findings support our hypothesis that BRIP1 promotes BC progression, ongoing structural and functional validation experiments in our laboratory aim to shed light on the exact mechanisms by which BRIP1‐downstream signalling promotes BC cell growth and cell invasion.

## CONFLICT OF INTEREST

The authors declare no conflicts of interest.

## AUTHOR CONTRIBUTIONS

**Balsam Rizeq:** Conceptualization (supporting); Data curation (lead); Formal analysis (supporting); Funding acquisition (supporting); Investigation (supporting); Methodology (lead); Validation (lead); Writing‐original draft (lead); Writing‐review & editing (supporting). **Saïd Sif:** Formal analysis (supporting); Methodology (equal); Supervision (equal); Writing‐review & editing (equal). **Gheyath K. Nasrallah:** Formal analysis (supporting); Writing‐review & editing (supporting). **Allal Ouhtit:** Conceptualization (lead); Data curation (equal); Formal analysis (equal); Funding acquisition (lead); Investigation (lead); Methodology (supporting); Project administration (lead); Supervision (lead); Validation (lead); Writing‐original draft (supporting); Writing‐review & editing (equal).

## Supporting information

Fig S1‐2

## Data Availability

All the data needed to evaluate the conclusion section are provided in the paper and/or the Supplementary Materials. Additional data related to this paper could be made available upon request from the corresponding author.
