# Supplementary material for: Novel role of BRCA1 interacting C‐terminal helicase 1 (BRIP1) in breast tumour cell invasion
Source: J Cell Mol Med. 2020 Sep 5;24(19):11477–88. doi: 10.1111/jcmm.15761 (PMC7576304; doi:10.1111/jcmm.15761)
Supplement: Supplementary file 1 — Fig S1‐2 [file JCMM-24-11477-s001.docx]

**Supplementary Data**

**
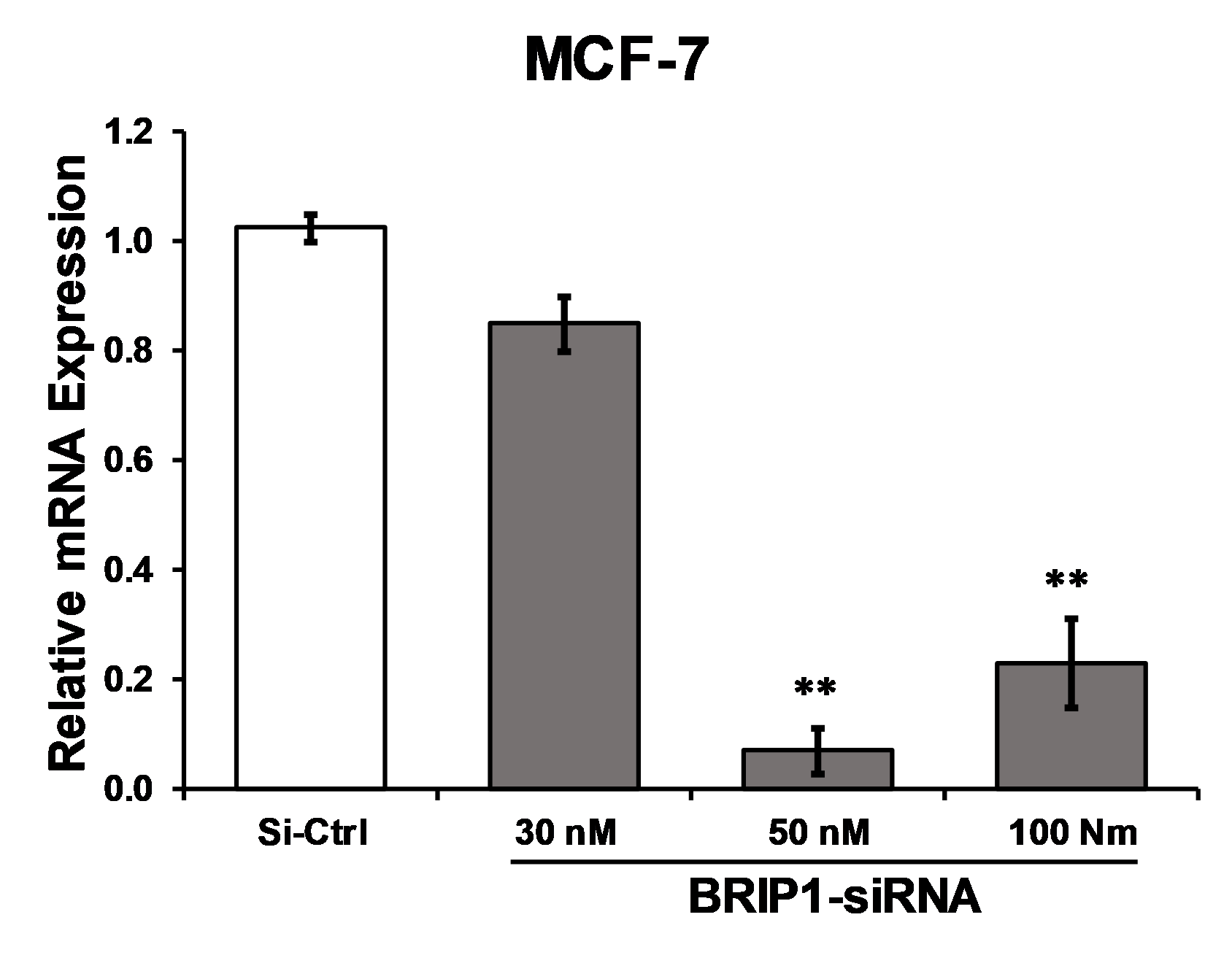
**

**Figure S1: Optimization of BRIP1-specific siRNAs concentration for efficient BRIP1 knockdown in MCF-7 cells.** Cells were transfected with 30, 50, and 100 nM siRNAs targeting *BRIP1* and si-Ctrl as described in methods. Total RNA was extracted at 72 h post-transfection, and TaqMan RT-qPCR assay determined the mRNA expression levels. All relative expression levels of *BRIP1* mRNA were normalized to *GAPDH* and relative to si-Ctrl. Mean values (n=3) ±SD of three experiments are shown; * *P*< 0.05, ** *P*< 0.01.


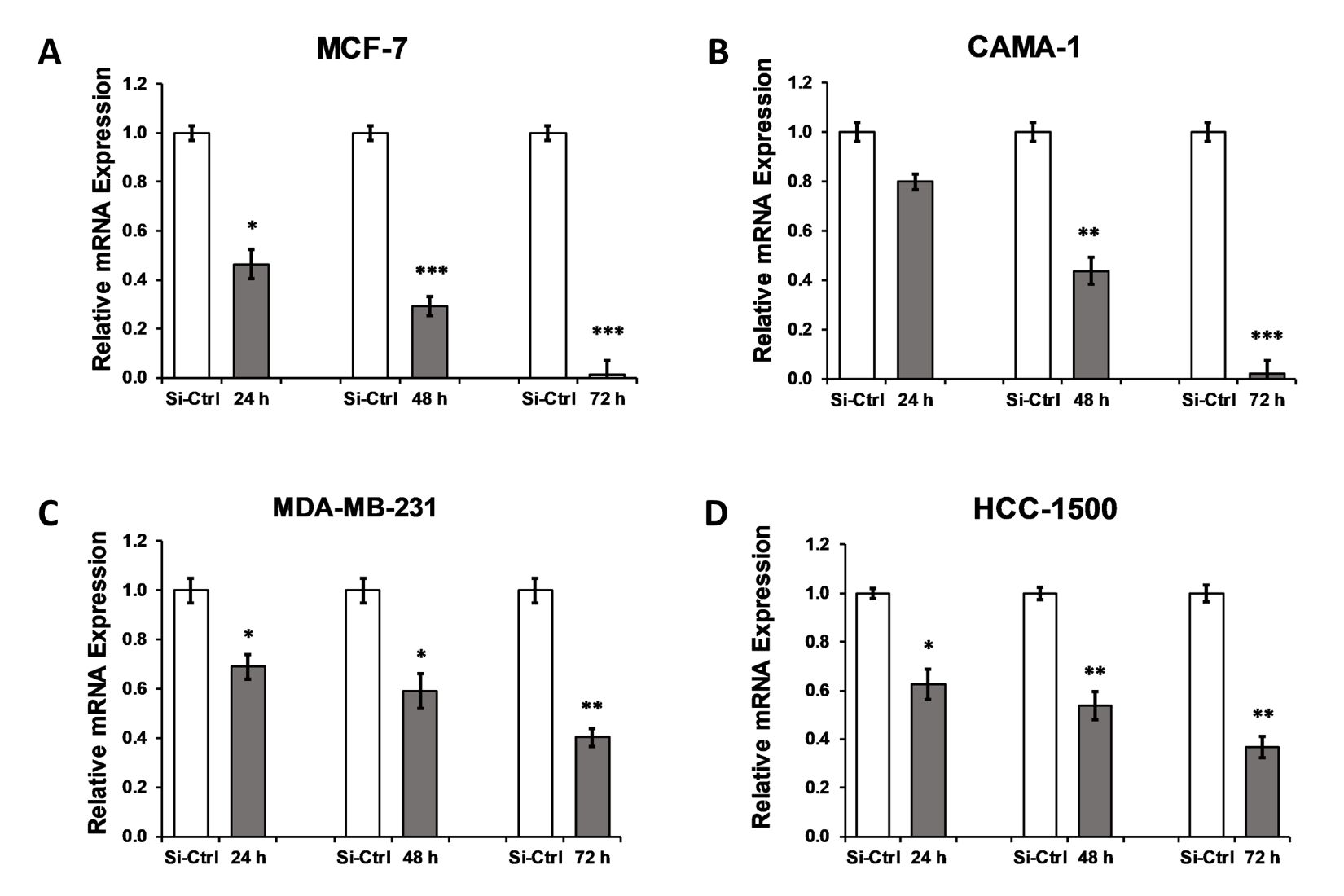


**Figure S2: Time optimization for efficient BRIP1 siRNAs-mediated knockdown in BC cells.** (A) MCF-7, (B) CAMA-1, (C) MDA-MB-231, and (D) HCC-1500 BC cells were transfected with 50 nM of siRNA targeting *BRIP1* and si-Ctrl as described in the methods. Total RNA was extracted post-transfection at 24, 48 and 72 h, and mRNA gene expression levels were determined by TaqMan RT-qPCR assay. All relative expression levels of *BRIP1* mRNA was normalized to *GAPDH* and relative to the si-Ctrl. Mean values ±SD (n=3) of three experiments are shown; * *P*< 0.05, ** *P*< 0.01, *** *P*< 0.001.
